# Unusually Prolonged Motor and Sensory Block Following Single Injection Ultrasound-Guided Infraclavicular Block With Bupivacaine and Dexamethasone

**DOI:** 10.5812/aapm.10583

**Published:** 2013-09-01

**Authors:** Mir Moussa Aghdashi, Kasra Dehghan, Shahram Shokohi, Shahrzad Shafagh

**Affiliations:** 1 Department of Anesthesiology, Urmia Medical Science University, Urmia, Iran

**Keywords:** Nerve Block, Dexamethasone

## Abstract

We present a case of unexpectedly prolonged motor and sensory block following a successful single injection ultrasound – guided infraclavicular block with bupivacaine (0.25%) and dexamethasone (8 mg). ultrasound guidance and safety measurement such as injection of the local anaesthetic at a slow rate and verifying that usual resistance was felt throughout the injection, has been applied. It took 42 hours for the block to go away. Although there was no evidence of neurologic injury but we should always be prepared to consider the possibility of nerve injury and take appropriate measures.

## 1. Background

Peripheral nerve blocks enjoy great importance in anaesthesia practice. They can provide safe and effective anaesthesia with long-lasting analgesia (1, 2). Brachial plexus block is a widely employed regional nerve block of the upper extremity. Various approaches have been described to block the brachial plexus. Infraclavicular approach represents a reliable and safe approach for the hand, forearm and the elbow surgeries. Different additives have been used as adjuvant with local anesthetics to achieve dense and prolonged block. Corticosteroids are believed to extend the duration of the nerve block. Dexamethasone has been used as an adjuvant to local anesthetics in peripheral nerve blocks (3, 4). We describe the case of a patient whose hand surgery was performed under ultrasound guided infraclavicular block using bupivacaine and dexamethasone. He had a very prolonged sensory and motor blockade for 42 hours. His recovery was uneventful.

## 2. Case Presentation

A 35 years-old male with left distal radius and ulna fracture was scheduled for closed reduction and percutaneous pinning. His Physical examination was normal. The patient did not have any past surgeries and didn’t take any medication. The patient was somewhat anxious about having surgery under general anesthesia and requested the regional anesthesia. Standard monitoring including non-invasive blood pressure recording, pulse oximetery and electrocardiography were applied and a single injection ultrasound guided infraclavicular block was implemented for the procedure. Midazolam 2 mg was given intravenously 10 minutes before the start of the block, and IV fentanyl (75 micg) before insertion of the needle was administered. We used a Honda HS – 2100 System and a 7.5-10 MHz linear ultrasound probe. The patient assumed a supine position and turned his head to the opposite side. 

With the probe placed in a parasagital plane medial and inferior to the coracoids process, we obtained a clear short-axis image of the axillary artery. The entry point of the needle was anesthetized with lidocaine (3 mL, 1%). Then, using an in-plane approach, a 22 gauge, 100-mm short bevel insulated needle (pajunk, UniPlex NanoLine) was advanced and placed posterior to the axillary artery, next to the posterior cord. After reassuring that the needle tip is in correct location, and confirming a negative aspiration for blood, we slowly injected bupivacaine (30 mL, 0.25%) and dexamethasone (8 mg), and then withdrew the needle. During the injection no adverse effect was noticed. Five minutes after the injection muscle coordination in the extremity was lost and the pain diminished substantially. These were indicative of an impending successful block. After 15 minutes the surgery was allowed to start. The surgical procedure (closed reduction and percutaneous pinning) lasted 40 minutes. After the end of surgery, the patient was monitored for half an hour in the PACU and then transferred to ward.

The next morning, we were informed that the patient had not recovered completely from the motor and sensory blockade. Examination of the patient proved the same. The patient’s condition was reassessed once more 24 hours after the performance of the block. No significant change in the patient’s condition was found; complete motor and sensory blockade was persistent. Despite that the patient had no complaint about pain or paresthesia during injection of the local anesthetic and that we did not notice any unusual resistance to injection, a complete motor and sensory block in C6, C7, and C8 had remained, and the possibility of damage to nerves emerged. A nerve conduction study was requested and until then a trained nurse from acute pain service was put in charge of evaluating the patient every hour. It was approximately thirty hours after the original injection that the nurse reported the patient had felt occasional stabbing pain in his fingers. Expecting that recovery would gradually come about, the nerve conduction study was postponed to a later time in the next morning. Complete recovery of motor and sensory functions required 42 hours. Second examination of the brachial plexus 24 hours later did not reveal any residual motor or sensory blockade. 

## 3. Discussion

Bupivacaine is a congener of mepivacaine. A butyl group on the piperidine ring has given it a longer duration of action. Longer duration of action together with its high quality sensory block, relative to its motor blockade, has established bupivacaine as the most commonly used local anesthetics (5, 6). Several studies have mentioned different mean duration of sensory blockade for bupivacaine. Bromage found that the longest mean duration of sensory block was 10.5 hours (7). Bupivacaine great affinity for fat has given the impression that its duration of action on peripheral nerves may be longer. Duration of action of nine hours in brachial plexus block and 17 hours in sciatic block (8), sensory block of 7.5 – 8.5 hours in ulnar nerve block (9) have been reported. A very wide range of duration of action from 5 to 16 hours has been proposed for bupivacaine (10). 

Many additives are used in conjunction with local anesthetics for regional and neuraxial anesthesia to quicken onset, prolong duration and enhance analgesia. Adjuvants include but are not limited to epinephrine, clonidine (11), opioids (12-16) midazolam (17), ketamine (18) and more recently dexamethasone (19, 20). We couldn’t find a reason that could explain this prolonged sensory and motor block. The dose of bupivacaine was less than what is routinely used for infraclavicular block and no epinephrine was used. The block was performed under ultrasound guide which permitted direct visualization of the target cords, the needle and axillary artery. Single injection ultrasound guided technique and lack of paresthesia and pain during procedure were further evidence that no direct trauma to the cords has happened. Due to the nature of the surgery, there was no need to apply a tourniquet. Total doses (75 mg) and concentration of the drug (0.25%) used in the procedure, did not justify the duration of motor and sensory block. It is believed that the incidence of neuronal injury in ultrasound guided regional anesthesia procedures is around 0.04%. (21, 22). The only thing unusual about the block was the use of dexamethasone as an adjuvant. The use of dexamethasone as an adjuvant to local anesthetics for peripheral nerve blocks is receiving interestly increasing. clinically Dexamethasone usage seems to lengthen the motor and sensory block time of peripheral nerve blocks. Debate still continues regarding the mechanism by which this effect occurs. Results of a few studies using steroids as adjuvant indicate that steroids have nerve block prolonging effect. Addition of steroid to local anaesthetic effectively and significantly prolongs the duration of analgesia and also anaesthesia and cause earlier onset of action (19, 23).

Practice of adding dexamethasone to local anesthetics for peripheral nerve block in our operation room is an occasional and arbitrary one and this is the first time we were faced with such a prolonged block. We cannot conclude that dexamethasone added to bupivacaine in this block, was the culprit behind this prolonged motor and sensory block. The results obtained from those studies also don’t support that dexamethasone could cause such a prolonged block. 

There are a few reports of prolonged blockade following seemingly flawless technique of performing block. Complete recovery in those case reports varied from 40 to 84 hours after the block (24-26). None of the papers have clearly stated the reason behind the long blockade. Injecting the local anesthetic too close to the nerves and chronic treatment with lithium has been proposed as reasons behind these unusually prolonged blocks. Luduena believed that causes of prolonged blockade are often unknown and if the duration is longer than 24 hours then probability of nerve damage should be considered (27).
